# Supplementary material for: US cities increasingly integrate justice into climate planning and create policy tools for climate justice
Source: Nat Commun. 2022 Sep 30;13:5763. doi: 10.1038/s41467-022-33392-9 (PMC9524340; doi:10.1038/s41467-022-33392-9)
Supplement: Supplementary file 1 — Supplementary Information [file 41467_2022_33392_MOESM1_ESM.pdf]

U.S. Cities Increasingly Integrate Justice into Climate Planning and Create Policy Tools for Climate Justice

SUPPLEMENTARY TABLES

Supplementary Table 1. List of analysed climate action plans.

Pop. Rank	City	State	Plan type*	Plan year	Plan title
1	New York	NY	CP	2019	A Livable Climate. OneNYC 2050: Building a Strong and Fair City
2	Los Angeles	CA	SP	2019	L.A.'s Green New Deal: Sustainable City pLAn
3	Chicago	IL	CP	2008	Chicago Climate Action Plan: Our City. Our Future
4	Houston	TX	CP	2020	Houston Climate Action Plan
6	Philadelphia	PA	EP	2018	Powering Our Future: A Clean Energy Vision for Philadelphia
7	San Antonio	TX	CP	2019	SA Climate Ready: A Pathway for Climate Action & Adaptation
8	San Diego**	CA	CP	2015	City of San Diego Climate Action Plan (+ 2019 Annual Report)
9	Dallas	TX	CP	2020	Dallas Comprehensive Environmental and Climate Action Plan
10	San Jose	CA	CP	2018	Climate Smart San Jose: A People-Centered Plan for a Low-Carbon City
11	Austin	TX	CP	2015	Austin Community Climate Plan
14	Columbus	OH	SP	2015	The Columbus Green Community Plan: Green Memo III
15	Charlotte**	NC	EP	2018	Charlotte Strategic Energy Action Plan (+ 2019 Annual Report)
16	San Francisco	CA	CP	2019	Focus 2030: A Pathway to Net Zero Emissions
17	Indianapolis	IN	CP	2019	Thrive Indianapolis
18	Seattle**	WA	CP	2013	Seattle Climate Action (+ 2018 Update)
19	Denver	CO	CP	2018	Denver 80x50 Climate Action Plan
20	Washington D.C.	DC	CP/EP	2018	Clean Energy DC: The District of Columbia Climate and Energy Action Plan
21	Boston	MA	CP	2019	City of Boston Climate Action Plan 2019 Update
24	Detroit	MI	CP	2017	Detroit Climate Action Plan
25	Oklahoma City	OK	SP	2020	Adaptokc: Adapting for a Healthy Future
26	Portland	OR	CP	2015	Climate Action Plan
28	Memphis	TN	CP	2020	Memphis Area Climate Action Plan
29	Louisville	KY	CP	2020	Louisville Greenhouse Gas Emissions Reduction Plan
30	Baltimore	MD	SP	2019	The 2019 Baltimore Sustainability Plan
31	Milwaukee	WI	SP	2013	ReFresh Milwaukee: City of Milwaukee Sustainability Plan 2013 - 2023
36	Sacramento	CA	CP	2015	General Plan Appendix B: Climate Action Plan Policies and Programs
37	Atlanta	GA	EP	2017	Clean Energy Atlanta: A vision for a 100% Clean Energy Future
38	Kansas City	MO	CP	2008	Climate Protection Plan: City of Kansas City, Missouri
41	Raleigh	NC	CP/EP	2012	A Roadmap to Raleigh's Energy Future
42	Miami	FL	CP	2008	MiPlan: City of Miami Climate Action Plan
45	Oakland	CA	CP	2020	Oakland 2030: Equitable Climate Action Plan
46	Minneapolis	MN	CP	2013	Minneapolis Climate Action Plan: A Roadmap to Reducing Citywide Greenhouse Gas Emissions
48	Tampa	FL	EP	2011	City of Tampa Energy Efficiency and Conservation Plan
50	New Orleans	LA	CP	2017	Climate Action for a Resilient New Orleans
53	Cleveland	OH	CP	2018	Cleveland Climate Action Plan 2018 Update: Building Thriving and Resilient Neighborhoods for All

57	Santa Ana	CA	CP	2015	Santa Ana Climate Action Plan
58	Riverside	CA	SP	2016	Economic Prosperity Action Plan and Climate Action Plan
62	Stockton	CA	CP	2014	City of Stockton Climate Action Plan
63	Saint Paul	MN	CP	2019	Saint Paul Climate Action & Resilience Plan
64	Cincinnati	OH	SP	2018	2018 Green Cincinnati Plan
65	St. Louis	MO	CP	2017	Climate Action & Adaptation Plan for the City of St. Louis
66	Pittsburgh	PA	CP	2018	City of Pittsburgh Climate Action Plan Version 3.0
67	Greensboro	NC	SP	2020	Sustainability Action Plan Greensboro, North Carolina
69	Anchorage	AK	CP	2019	Anchorage Climate Action Plan
70	Plano	TX	CP	2020	City of Plano Cleaner Air & Reduced Emissions Strategy
71	Orlando	FL	SP	2018	2018 Community Action Plan
73	Newark	NJ	SP	2013	The City of Newark Sustainability Action Plan
74	Durham	NC	CP	2007	City of Durham & Durham County Greenhouse Gad and Criteria Air Pollutant Emissions Inventory and Local Action Plan for Emission Reductions
75	Chula Vista	CA	CP	2017	Chula Vista Climate Action Plan
78	St. Petersburg	FL	SP	2019	Integrated Sustainability Action Plan
82	Madison	WI	EP	2018	100% Renewable Madison: Achieving 100% Renewable Energy & Zero Net Carbon for City Operations & Leading the Community
85	Reno	NV	SP/CP	2019	City of Reno Sustainability & Climate Action Plan
90	Winston-Salem	NC	CP	2008	Winston-Salem Greenhouse Gas Inventory and Local Action Plan to Reduce Emissions
91	Chesapeake	VA	SP	2009	Chesapeake Sustainability Plan
92	Norfolk	VA	CP	2019	The City of Norfolk Climate Action Plan
93	Fremont	CA	CP	2012	City of Fremont Climate Action Plan
97	Richmond	VA	SP	2011	City of Richmond RVAgreen: A Roadmap to Sustainability
98	Boise City	ID	EP	2019	Boise’s Energy Future: A Community-wide Energy Plan

* CP: Climate plan; SP: Sustainability plan; EP: Energy plan. ** Cities with more than one analysed document.

Supplementary Table 2. Final coding protocol.

Main theme	Sub-theme	Main category	Sub-category
1. Distributional justice	A. Awareness	A1. Does the plan acknowledge that climate mitigation will procedure benefits that can advance or hinder justice? If yes, what benefits are considered?	A1.1. Health benefits<br>A1.2. Economic benefits<br>A1.3. Environmental benefits<br>A1.4. Technological benefits<br>A1.5. Resilience benefits<br>A1.6. Other
		A2. Does the plan acknowledge that climate mitigation will produce costs that can unequally distributed around the city? If yes, what costs are considered?	A2.1. Health costs<br>A2.2. Economic costs<br>A2.3. Environmental costs<br>A2.4. Technological costs<br>A2.5. Resilience costs<br>A2.6. Other
	B. Policy actions	B1. Does the plan include provisions to ensure that the benefits and costs of climate mitigation are distributed equitably? If yes, what provisions are included?	B1.1. Targeting or prioritising vulnerable groups and their needs<br>B1.1.1 Targeted workforce training<br>B1.2. Providing special benefits to vulnerable groups<br>B1.3. Avoiding or minimising burdens to vulnerable groups<br>B1.4. Improving access to benefits of climate action<br>B1.5. Justice or equity-oriented policy development<br>B1.5.1. Equity tools to design or implement policies<br>B1.5.2. Justice or equity as a policy design criterion<br>B1.5.3. Other systematic strategy<br>B1.6. Other
	C. Policy evaluation	C1. Does the plan include provisions to monitor or evaluate the distribution of climate mitigation outcomes? If yes, how?	C1.1. Justice indicators<br>C1.2. Justice evaluation tool
2. Procedural justice	D. Engagement and participation	D1. Have local community actors been engaged in the planning, implementation, or evaluation of mitigation policies? If yes, what groups have been represented?	D1.1. Advocacy groups<br>D1.1.1 Climate or environmental justice<br>D1.1.2 Racial justice (not climate or environmental)<br>D1.1.3 Gender justice<br>D1.1.4 Economic justice<br>D1.1.5 Religious interests<br>D1.1.6 Other interests<br>D1.2. Vulnerable groups<br>D1.3. General public<br>D1.4. Private sector<br>D1.5. Educational institutions<br>D1.6. Other
		D2. In which stage of the policy process have local community actors been engaged?	D2.1. Policy planning and design<br>D2.1.1. Justice-oriented steering committee<br>D2.1.2. Justice-oriented advisory committee<br>D2.1.3. Justice-oriented working group<br>D2.2. Policy implementation<br>D2.2.1. Justice-oriented implementation bodies<br>D2.2.2. Justice-oriented partnerships.<br>D2.3. Policy evaluation
	E. Access to information	E1. Does the plan include provisions to facilitate access to information related to climate change or climate mitigation policy? If yes, how is access to information provided?	E1.1. General outreach and education about climate change and mitigation<br>E1.2. General outreach about available city policies, programs, and engagement opportunities<br>E1.3. Outreach to the private sector about city policies, programs, and technical assistance opportunities

			E1.4. Targeted outreach to vulnerable populations about available city policies, programs, and engagement opportunities<br>E1.5. Other
		E2. Has the plan been translated to other languages? If yes, to what extent?	E2.1. The plan was completely translated to one or more languages.<br>E2.2. A summary of the plan was translated to one or more languages.
3. Justice as recognition	F. Historical or structural inequities	F1. Does the plan recognise the historical or structural roots of inequity in the city? If yes, what forms of inequities are recognised?	F1.1. Racial inequities<br>F1.2. Economic inequities<br>F1.3. Gender inequities<br>F1.4. Age inequities<br>F1.5. Other inequities
	G. Current inequities	G1. Does the plan recognise the intersection between climate mitigation and the current, everyday challenges of local residents? If yes, what challenges are articulated?	G1.1. Health challenges<br>G1.2. Economic challenges<br>G1.3. Environmental challenges<br>G1.4. Technological challenges<br>G1.5. Resilience challenges<br>G1.6. Other
		G2. Does the plan recognise the current inequities experienced in the city? If yes, what forms of inequities are recognised?	G2.1. Racial inequities<br>G2.2. Economic inequities<br>G2.3. Gender inequities<br>G2.4. Age inequities<br>G2.5. Other inequities
		G3. Does the plan include commitments to address or rectify historical and current inequities experienced in the city? If so, how?	G3.1. Targeting or prioritising vulnerable groups and their needs<br>G3.2. Providing special benefits to vulnerable groups<br>G3.3. Avoiding or minimising burdens to vulnerable groups<br>G3.4. Improving access to benefits of climate action<br>G3.5. Justice or equity-oriented policy development<br>G3.6. Facilitating access to information<br>G3.7. Actor engagement<br>G3.8. Other strategies
	H. Recognition of Indigenous communities	H1. Does the plan recognise differences in the value that nature has over the cultural or individual identity of Indigenous or Native American communities? If so, how?	
4. Justice in climate mitigation sectors	I. Climate mitigation sectors	I1. What sectors are recognised and included as climate mitigation in the plan?	I1.1. Clean energy<br>I1.2. Energy efficiency<br>I1.3. Land use and transport<br>I1.4. Electric vehicles<br>I1.5. Urban greening<br>I1.6. Waste<br>I1.7. Food<br>I1.8. Water<br>I1.9. Air quality
		I2. In which sectors did climate mitigation policies included notions of justice?	I2.1. Clean energy<br>I2.2. Energy efficiency<br>I2.3. Land use and transport<br>I2.4. Electric vehicles<br>I2.5. Urban greening<br>I2.6. Waste<br>I2.7. Food<br>I2.8. Water<br>I2.9. Air quality
5. Key definitions	J. Justice	J1. Is justice defined in the plan? If so, how?	
	K. Equity	K1. Is equity defined in the plan? If so, how?	
6. Key sections where justice is articulated	L. Key articulations of justice	L1. Are justice or equity included in one of the following features or sections of the plan?	L1.1. Goal or objective<br>L1.2. Vision<br>L1.3. Principles or values<br>L1.4. Mayor’s letter<br>L1.5. Letter from other city official

Supplementary Table 3. Key criteria to categorise cities by their level of engagement with justice in policy action.

**Cities that do not articulate justice as a core feature of climate action**	**Cities articulating justice as an aspiration**	**Cities explicitly planning for justice**
Cities in this category do not demonstrate that they have included justice or equity as core feature of their plan. This means that cities do not articulate justice or equity as a goal, objective, vision, principle, or value of their plan, and they do not demonstrate that they have systematically embedded justice into the development of their policy actions.	Cities in this category must demonstrate that justice or equity are part of the plan's goals, objectives, vision, principles, or values. This may be expressed in any section of the plan, including the Mayor's letter and/or other city official's letter.<br><br>Cities must thus meet one or more of the following criteria:<br><br>L1.1. Goal or objective<br>L1.2. Vision<br>L1.3. Principles or values<br>L1.4. Mayor's letter<br>L1.5. Letter from other relevant city official<br><br>Cities in this category do not demonstrate that they have systematically embedded justice into the development of their policy actions. This means that cities do not meet the following key criteria:<br><br>B1.5. Justice or equity-oriented policy development<br>B1.5.1. Equity tools to design or implement policies<br>B1.5.2. Justice or equity as a policy design criterion<br>B1.5.3. Other systematic strategy<br><br>C1.2. Justice evaluation tool	Cities in this category must demonstrate that they have systematically embedded justice into the development of their policy actions. Key criteria to consider are:<br><br>B1.5. Justice or equity-oriented policy development<br>B1.5.1. Equity tools to design or implement policies<br>B1.5.2. Justice or equity as a policy design criterion<br>B1.5.3. Other systematic strategy<br><br>C1.1. Justice indicators<br><br>C1.2. Justice evaluation tool<br><br>D2.1. Policy planning and design<br>D2.1.1. Justice-oriented steering committee<br>D2.1.2. Justice-oriented advisory committee<br>D2.1.3. Justice-oriented working group<br><br>D2.2. Policy implementation<br>D2.2.1. Justice-oriented implementation bodies<br>D2.2.2. Justice-oriented partnerships

Supplementary Table 4. Cities articulating justice indicators for policy evaluation.

**City name**	**Description of justice-oriented indicators**
Baltimore, MD	The city reports that it will monitor its ability to advance equity and sustainability goals. This will be done in three ways: **(1) annual reporting**: annual reports will include “thoughtful indicators” that evaluate city’s efforts and effectiveness of acting in a “racially equitable way”; **(2) annual open house**: the city will check in every year with its residents to identify new strategies and actions towards equity; **(3) periodic updates**: at least every three years, the city will update strategies, set new benchmarks, and identify new or refined metrics. The indicators are not explicitly included in this version of the plan.
Charlotte, NC	The city reports that the Charlotte Resilient Delivery Team (CREDIT) will continuously monitor all projects as they are being implemented to ensure “an equitable approach” is taken. The indicators are not explicitly included in this version of the plan.
Dallas, TX	The city reports that all programs and initiatives will be evaluated based on performance metrics that include “equity indicators”. The plan includes a table of draft metrics that may be potentially used for this purpose.
Minneapolis, MN	The city reports that it will include “equity indicators” to measure whether the plan’s strategies, investments, and emissions and energy burden reductions are experienced equitably across neighborhoods, income classes, and races. The indicators are not explicitly included in this version of the plan.
Portland, OR	The city reports that it will develop “climate-equity metrics” to track the degree to which Portland’s Equity Considerations are integrated into decision-making processes and implementation of the plan, as well as progress on “equitable outcomes”. The indicators are not explicitly included in this version of the plan, but the city articulates that metrics should measure distributional, procedural and structural equity.
San Antonio, TX	The city reports that it will identify and track “key climate equity indicators” as part of the plan’s implementation. The indicators are not explicitly included in this version of the plan.
San Diego, CA	The city developed the Climate Equity Index to measure the “equity impacts” of the plan. The index incorporates 35 environmental and socioeconomic indicators and calculates a climate equity score for each of the census tracks intersecting the city.
St. Petersburg, FL	The city reports that one of its “early actions” for implementation will be to build on existing performance tracking tools to measure progress on “equity”, among other topics. The indicators are not explicitly included in this version of the plan.

Supplementary Table 5. Descriptive statistics and description of dependent and predictor variables.

Variable	Min - Max	Mean	Standard Deviation	Description
**Dependent variables**				
City Category	1 – 3	2.0344	0.8158	3: City is explicitly planning for justice (*n* = 20).<br>2: City articulates justice as an aspiration of their climate plan (*n* = 20).<br>1: City does not incorporate justice into their climate plan (*n* = 18).
**Predictor variables**				
Climate plan characteristics				
After 2017	0 – 1	0.6207	0.4895	1: Climate plan published after 2017.<br>0: Climate plan published before 2017.
Plan Type	0 – 1	0.6552	0.4795	1: Climate plan is focused on climate change.<br>0: Climate plan covers climate change, but it is not only focused on climate change.
First Plan	0 – 1	0.6552	0.4795	1: Climate plan is the city's first plan.<br>0: Climate plan is a revised or updated plan.
Engagement	0 – 1	0.9138	0.2831	1: City mentions engaging with local community members for the climate plan.<br>0: City does not mention engaging with local community members for the climate plan.
Local city characteristics				
Population	0 – 1	0.4655	0.5032	1: City population > 500,000.<br>0: City population < 500,000.
Median Household Income	0 – 1	0.4138	0.4968	1: MHI > sample mean $61,532.<br>0: MHI < sample mean $61,532.
Poverty	4.30 – 35.0	17.50	5.84	Persons in poverty 2019, percentage.
Gini Index	0.4004 – 0.5815	0.4918	0.0409	Gini Index of income inequality 2015-2019.
People of color	16.80 – 90.60	58.57	16.81	Population who did not identify as "White alone, not Hispanic or Latino", 2019. This includes African American, Native American, Native Hawaiian and Pacific Islander, Asian, Hispanic or Latino, or two or more races. Percentage.
Median Age	30.70 – 42.90	34.74	2.29	Median age of population, 2015-2019.
Bachelor Attainment	15.00 – 64.00	38.02	11.73	Persons with a bachelor's degree or higher 2015 – 2019, percentage of persons age 25+ years.
Government Type	0 – 1	0.5517	0.5017	1: City has a "strong mayor" government type.<br>0: City does not have a "strong mayor" government type.
Democrat Vote	38.70 – 92.80	64.50	12.23	Democrat vote share for the 2016 presidential election in the city's main County, percentage.
Climate Network	0 – 1	0.6724	0.4734	1: City is a member of Global Covenant of Mayors.<br>0: City is not a member of Global Covenant of Mayors.
Coastal City	0 – 1	0.3621	0.4848	1: City is geographically located by the coast.<br>0: City is not geographically located by the coast.
Legacy City	0 – 1	0.2586	0.4417	1: City has been classified as a "legacy city".<br>0: City has not been classified as a "legacy city".
