# Supplementary material for: US cities increasingly integrate justice into climate planning and create policy tools for climate justice
Source: Nat Commun. 2022 Sep 30;13:5763. doi: 10.1038/s41467-022-33392-9 (PMC9524340; doi:10.1038/s41467-022-33392-9)
Supplement: Supplementary file 3 — Reporting Summary [file 41467_2022_33392_MOESM3_ESM.pdf]

nature portfolio

Corresponding author(s): Claudia Viridiana Diezmartinez Peregrina

Last updated by author(s): Aug 20, 2022

Reporting Summary

Nature Portfolio wishes to improve the reproducibility of the work that we publish. This form provides structure for consistency and transparency in reporting. For further information on Nature Portfolio policies, see our Editorial Policies and the Editorial Policy Checklist.

Statistics

For all statistical analyses, confirm that the following items are present in the figure legend, table legend, main text, or Methods section.

n/a	Confirmed	
☐	☒	The exact sample size (*n*) for each experimental group/condition, given as a discrete number and unit of measurement
☒	☐	A statement on whether measurements were taken from distinct samples or whether the same sample was measured repeatedly
☐	☒	The statistical test(s) used AND whether they are one- or two-sided<br>*Only common tests should be described solely by name; describe more complex techniques in the Methods section.*
☐	☒	A description of all covariates tested
☐	☒	A description of any assumptions or corrections, such as tests of normality and adjustment for multiple comparisons
☐	☒	A full description of the statistical parameters including central tendency (e.g. means) or other basic estimates (e.g. regression coefficient) AND variation (e.g. standard deviation) or associated estimates of uncertainty (e.g. confidence intervals)
☐	☒	For null hypothesis testing, the test statistic (e.g. *F*, *t*, *r*) with confidence intervals, effect sizes, degrees of freedom and *P* value noted<br>*Give P values as exact values whenever suitable.*
☒	☐	For Bayesian analysis, information on the choice of priors and Markov chain Monte Carlo settings
☒	☐	For hierarchical and complex designs, identification of the appropriate level for tests and full reporting of outcomes
☒	☐	Estimates of effect sizes (e.g. Cohen's *d*, Pearson's *r*), indicating how they were calculated

Our web collection on statistics for biologists contains articles on many of the points above.

Software and code

Policy information about availability of computer code

Data collection	No software was used for data collection.
Data analysis	We used NVivo 12 Pro software for the qualitative coding of urban climate action. We used R for the ordinal logistic regression analysis.<br><br>The data used and generated in this study (city climate action plans, qualitative content analysis, socio-demographic data, logistic regression analysis) have been deposited in an open repository: https://doi.org/10.5281/zenodo.7008298

For manuscripts utilizing custom algorithms or software that are central to the research but not yet described in published literature, software must be made available to editors and reviewers. We strongly encourage code deposition in a community repository (e.g. GitHub). See the Nature Portfolio guidelines for submitting code & software for further information.

Data

Policy information about availability of data

All manuscripts must include a data availability statement. This statement should provide the following information, where applicable:

- Accession codes, unique identifiers, or web links for publicly available datasets
- A description of any restrictions on data availability
- For clinical datasets or third party data, please ensure that the statement adheres to our policy

The data used and generated in this study (city climate action plans, qualitative content analysis, socio-demographic data, logistic regression analysis) have been deposited in an open repository: https://doi.org/10.5281/zenodo.7008298

Field-specific reporting

Please select the one below that is the best fit for your research. If you are not sure, read the appropriate sections before making your selection.

☐ Life sciences ☒ Behavioural & social sciences ☐ Ecological, evolutionary & environmental sciences

For a reference copy of the document with all sections, see nature.com/documents/nr-reporting-summary-flat.pdf

Behavioural & social sciences study design

All studies must disclose on these points even when the disclosure is negative.

Study description	Mixed-methods approach using qualitative content analysis of urban climate action plans and ordinal logistic regression.
Research sample	Our research sample is composed of the most recent climate action plan adopted by each of the 100 largest cities in the US. The sample of cities included in our study was defined according to the US Census Bureau 2019 population estimates. For each city, we selected the most recently adopted climate action plan that fit our definition as of June 2021.<br><br>We focus on large cities because (a) these urban areas are more likely to have more diverse populations that experience relatively pronounced poverty and income disparities, and (b) their governments are more likely to have more resources and capacities to undergo complex climate planning processes that incorporate equity. Moreover, focusing on large cities enables us to compare our findings across previous studies, most of which examine climate planning in large cities. We also focus only in the most recent climate plans to capture the latest and most novel conceptualizations of climate justice in urban climate action planning in the U.S.<br><br>Since we focus only on the largest cities in the U.S., our sample may not be representative of efforts occurring in smaller municipalities across the U.S. or other countries.<br><br>All climate plans are publicly available online through each city's website.
Sampling strategy	The sample was determined according to set criteria: a) climate plan must be adopted by the city government from one of the 100 largest cities in the US and b) climate plan must include explicit policies for climate mitigation (see explanation in Data exclusions).
Data collection	We collected plans through targeted internet searches in Google (e.g., “city name” + “climate action plan”), city government websites, and the Local Government Climate and Energy Goals database developed by the American Council for an Energy-Efficient Economy (ttps://database.aceee.org/city/local-government-energy-efficiency-goals). All climate plans used are publicly available in each city's website and contained in files deposited in our open repository.<br><br>Data used for the regression analysis was obtained from the 2019 U.S. Census estimates and the 2015-2019 American Community Survey and extracted through R's function "get_acs()". Our data availability statement has a link to a repository that includes the data collected through this strategy and used for the regression analysis.<br><br>Researchers were not blinded for hypothesis testing.
Timing	Data collection started on September 2020 and ended in June 2021.
Data exclusions	For each city, we selected the most recently adopted climate action plan that fit our definition as of June 2021. We define climate action plan as any formal local planning document adopted by a city government that explicitly addresses multiple sectors of climate mitigation. This definition includes climate plans exclusively focused on mitigation, climate plans integrating mitigation and adaptation or resilience, as well as sustainability and energy plans with chapters or sections explicitly dedicated to mitigation. We excluded city plans that are only focused on climate adaptation or resilience, plans that are written by state or regional entities, and plans that are written by local entities (e.g., local non-profits, universities) but not formally adopted by city governments.<br><br>Several cities in our sample had published multiple plans over the past decade. In cases where the most recent plan updated or superseded earlier plans, we reviewed only the most recent plan. However, in cases where the most recent plan complemented an earlier plan, we reviewed both the most recent and previous versions of the plan. In total, we found that 58 out the 100 largest US city had an eligible climate action plan to include in our analysis.
Non-participation	No participants were required in this study.
Randomization	The sample of climate plans analyzed in this study was not randomized, as the goal of the study was to analyze the 100 largest cities in the U.S. Such sample is, de facto, not random. Our goal was not to generate a randomized and representative sample of cities, but rather capture the efforts of large cities, which are commonly recognized as leaders in urban climate action.

Reporting for specific materials, systems and methods

We require information from authors about some types of materials, experimental systems and methods used in many studies. Here, indicate whether each material, system or method listed is relevant to your study. If you are not sure if a list item applies to your research, read the appropriate section before selecting a response.

Materials & experimental systems

n/a	Involved in the study
☒	☐ Antibodies
☒	☐ Eukaryotic cell lines
☒	☐ Palaeontology and archaeology
☒	☐ Animals and other organisms
☒	☐ Human research participants
☒	☐ Clinical data
☒	☐ Dual use research of concern

Methods

n/a	Involved in the study
☒	☐ ChIP-seq
☒	☐ Flow cytometry
☒	☐ MRI-based neuroimaging
